# A common genetic factor underlies hypertension and other cardiovascular disorders

**DOI:** 10.1186/1471-2261-4-20

**Published:** 2004-11-01

**Authors:** Frances MK Williams, Lynn F Cherkas, Tim D Spector, Alex J MacGregor

**Affiliations:** 1 Twin Research and Genetic Epidemiology Unit, St Thomas' Hospital, London SE1 7EH, UK; 2 Department of Medicine, University of East Anglia, Norwich NR4 7TJ, UK

## Abstract

**Background:**

Certain conditions characterised by blood vessel occlusion or vascular spasm have been found to cluster together in epidemiological studies. However the biological causes for these associations remain controversial. This study used a classical twin design to examine whether these conditions are linked through shared environmental exposures or by a common underlying genetic propensity to vasospasm.

**Methods:**

We investigated the association between hypertension, migraine, Raynaud's phenomenon and coronary artery disease in twins from a national register. Phenotype status was determined using a questionnaire and the genetic and environmental association between phenotypes was estimated through variance components analysis.

**Results:**

Responses were obtained from 2,204 individuals comprising 525 monozygotic and 577 dizygotic pairs. There was a significant genetic contribution to all four traits with heritabilities ranging from 0.34 to 0.64. Multivariate model-fitting demonstrated that a single common genetic factor underlies the four conditions.

**Conclusions:**

We have confirmed an association between hypertension, migraine, Raynaud's phenomenon and coronary artery disease, and shown that a single genetic factor underlies them. The demonstration of a shared genetic factor explains the association between them and adds weight to the theory of an inherited predisposition to vasospasm.

## Background

A number of conditions characterised by blood vessel occlusion and/or vascular spasm have been found to be associated in both clinical and epidemiological studies. These include hypertension (HPT), Raynaud's phenomenon (RP), migraine (MIG) and coronary artery disease (CAD) [1-5]. Whether these associations are the consequences of a shared environmental risk factor or represent an underlying propensity to develop the conditions through a common biological mechanism remains controversial. The exploration of the genetic and environmental relationships underlying these conditions is one approach to resolving the biological basis for the association. We have examined the association between these phenotypes in a classical twin study conducted in a large sample. This approach allows us to assess whether the association between HPT, RP, MIG and CAD is explained by a common genetic or environmental aetiology.

## Methods

### Subjects and methods

Subjects for this study were twins enlisted with the St Thomas' UK Adult Twin Registry [6]. These monozygotic (MZ) and dizygotic (DZ) twin volunteers have been recruited since 1992 using twin registers and successive national media campaigns. For historical reasons most enrolled twins are female. This well studied population is sent regular questionnaires for self-completion concerning a wide range of health issues. Questions relating to HPT, MIG, RP and CAD respectively were contained within large questionnaires sent to the twins in 1998 and 2000. The questions were non-consecutive and respondents were unaware of the hypothesis being tested. The questionnaires also included standard questions relating to zygosity assignment [7]. In addition, fifty-four percent of the respondents had their zygosity assigned with certainty by multiplex DNA fingerprinting using variable tandem repeats on samples taken on previous attendances at the Twin Unit.

### Classification of HPT, RP, MIG AND CAD

Classification of the traits HPT, RP, MIG and CAD was based on standard, validated criteria where possible. HPT was classified by asking about a doctor's diagnosis of "high blood pressure" when not pregnant. Questions to determine presence of migraine were based on the UCSD Migraine Questionnaire [8] (including at least five episodes of unilateral, pulsating headache over the previous year, duration four to seventy-two hours, noise and light sensitivity). RP was classified by confirming digital sensitivity to cold and required the respondent to have had recurrent episodes of colour change involving at least two colours [9]. CAD was classified either by respondents having a doctor's diagnosis of heart disease or angina, a previous heart attack or heart operation; or by answering affirmatively to questions taken from the Rose Angina questionnaire [10].

### Analysis

Traits were defined categorically as present or absent using the definitions listed above. Phenotypic associations between the four traits were assessed using odds ratios and 95% confidence intervals (CI) corrected to take into account the paired nature of the data. The odds ratios were adjusted for possible confounding by age, smoking and body mass index (BMI). Evidence for a genetic contribution to each trait was examined by estimating casewise concordance [11]. This measures the probability that the co-twin of an affected twin also expresses the trait. Higher casewise concordance in MZ pairs compared to DZ twin pairs indicates a genetic effect.

Where data suggested a genetic influence, a quantitative measure of genetic contribution was estimated using structural equation modelling (Mx software [12]). This standard approach to twin analysis assumes an underlying model in which the observed phenotypic correlation among twins is explained by latent additive genetic influences (A) (which have a correlation of 1 in MZ twins and 0.5 in DZ twins), common environmental influences (C) (having a correlation of 1 in both MZ and DZ twins) and the random environment (E) (uncorrelated among twins). Analysis proceeds on the assumption that the observed categorical phenotype is accounted for by a continuous underlying liability to the trait [13]. Correlation in underlying liability is used as the basis for measuring the association between variables in the modeling. The significance of shared genetic and environmental factors was tested by stepwise deletion in a sequence of models containing the variance components (A, C and E) and assessing the deterioration in chi-squared for the fit of the model. Heritability was estimated from the size of the additive genetic contribution to the final selected model.

Modeling was then extended to consider the genetic and environmental associations between all four variables simultaneously [13]. Three multivariate models were considered:-

**Figure 1:**
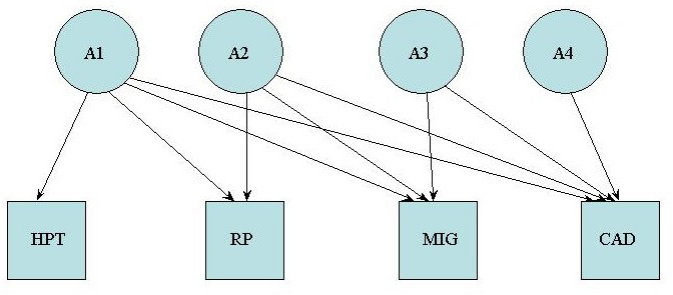
The Cholesky AE model. Diagram representing the Cholesky AE model, in which additive genetic (A) effects are shown loading on to the four traits: the unique environment (E) would load similarly

**Figure 2:**
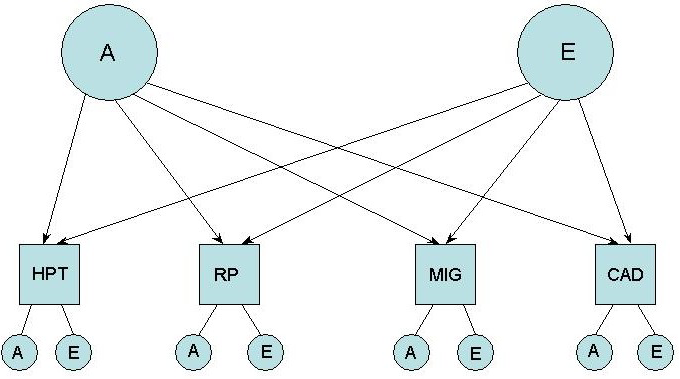
The independent AE pathway model. Diagram of the independent AE pathway, in which a single genetic and single unique environment factor loads on to each of the four traits, as well as factors specific to each trait

**Figure 3:**
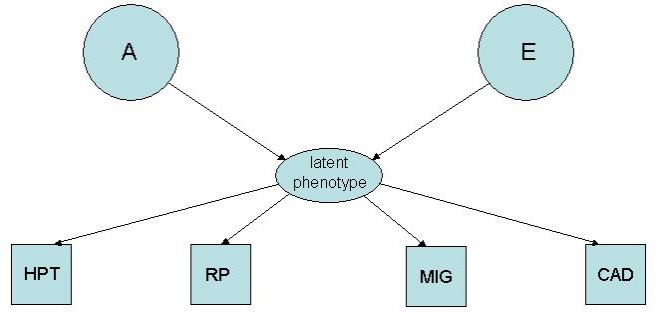
The common pathway model. Diagram of the common factor pathway in which single genetic and unique environment factors load on to the traits via a phenotypic latent variable

(1) the Cholesky decomposition (Figure 1) which includes four independent genetic and environmental factors. The first factor loads on all the traits, the second factor loads on all traits except the first, the third loads on all traits except the first two etc. This provides the fullest potential explanation of the data

(2) the independent pathway model (Figure 2) is a submodel of the Cholesky model and considers the data to be explained by a single shared genetic and shared environmental factor

(3) the common pathway model (Figure 3) which considers a single shared latent phenotype, determined in turn by latent genetic and environment factors.

The suitability of the common and independent pathway models may be determined by comparing the model's Akaike information criterion (AIC) with that of the fullest model, the Cholesky. The AIC represents the balance between model fit and the number of parameters (parsimony), with lower values of AIC indicating the most suitable model. Parameter estimates from the most appropriate model were used to calculate the genetic and environmental correlation between variables.

## Results

Complete data for analysis were available from 1,102 pairs of female twins, comprising 525 MZ and 577 DZ pairs. The mean (± SD) age of the twins was 48.5 (± 8.2) years. The prevalence of each trait is shown in Table 1.

**Table 1:** Prevalence of the traits by zygosity and the p value of the difference between zygosities

trait	MZ prevalence (%) n = 1050	DZ prevalence (%) n = 1154	p
HPT	131 (12.5)	181 (15.7)	0.052
RP	111 (10.6)	131 (11.4)	0.584
MIG	264 (25.1)	266 (23.1)	0.290
CAD	71 (6.8)	76 (6.6)	0.873

n represents number of twins, MZ monozygotic, DZ dizygotic, HPT hypertension, RP Raynaud's phenomenon, MIG migraine, CAD coronary artery disease

Four of the six combinations of traits showed significant phenotypic association after adjusting for age, smoking and BMI (odds ratios, Table 2). HPT was clearly not associated with RP but there was a suggestion of association of HPT with migraine (significantly so if adjusted for age and smoking only, data not shown). Since smoking status, age and BMI did not influence the size of the odds ratios, these variables were not included in the multivariate modeling. Concordance data and the results of univariate modeling confirmed a significant heritable component to all 4 traits (Table 3). Multivariate model fitting showed that for each of the three models tested, a model containing genetic (A) and unique environmental (E) factors provided the best explanation of the data (Table 4, in bold): incorporation of a shared environmental factor (C) offered no significant improvement in the fit of any model. The independent AE pathway model (Figure 2) offered the best explanation of the data, suggesting that a single common genetic factor loads on the four traits HPT, RP, migraine and CAD. A unique environmental factor loads similarly on the traits. Using this model, genetic and unique environmental correlations between the four traits were calculated (Table 5). Overall, the genetic correlations were greater than the environmental correlations, suggesting a greater role for the genetic factor than the unique environmental factor.

**Table 2:** Odds ratios (95% confidence interval) of unadjusted and adjusted (for age, smoking and BMI) cross-trait associations

	HPT	RP	MIG	CAD
HPT				
adj				
RP	0.88 (0.59–1.31)			
adj	0.99 (0.64–1.54)			
MIG	1.31 (0.99–1.73)	1.62 (1.21–2.17)		
adj	1.31 (0.95–1.80)	1.68 (1.23–2.3)		
CAD	2.85 (1.96–4.16)	2.03 (1.29–3.17)	2.19 (1.53–3.13)	
adj	2.41 (1.56–3.73)	2.27 (1.42–3.65)	1.77 (1.19–2.63)	

Calculation of odds ratios included a correction factor for familial clustering. Unadjusted odds ratios are shown above and adjusted below, for each combination of phenotypes. BMI represents body mass index, HPT hypertension, RP Raynaud's phenomenon, MIG migraine, CAD coronary artery disease.

**Table 3:** Casewise concordance rates by zygosity and heritability estimates of the four traits

	twin type	total pairs	discordant pairs (+/-)	concordant pairs with trait (+/+)	casewise concordance (95% CI)	heritability (95% CI)
**HPT**	MZ	525	73	29	0.44 (0.34–0.55)	0.64 (0.49–0.79)
	DZ	577	135	23	0.25 (0.17–0.34)	
**RP**	MZ	525	75	18	0.32 (0.21–0.44)	0.46 (0.30–0.63)
	DZ	577	111	10	0.15 (0.07–0.24)	
**MIG**	MZ	525	144	60	0.46 (0.38–0.53)	0.43 (0.30–0.55)
	DZ	577	186	40	0.30 (0.23–0.37)	
**CAD**	MZ	525	55	8	0.23 (0.10–0.35)	0.34 (0.13–0.55)
	DZ	577	72	2	0.05 (0.00–0.12)	

Casewise concordance rates (95% confidence intervals, CI) are shown for each trait by zygosity. An estimate of trait heritability (95% CI) is also given.

HPT represents hypertension, RP Raynaud's phenomenon, MIG migraine, CAD coronary artery disease.

**Table 4:** Results of the model fitting: comparison of the 3 models

	**model**	**chi**^2^	**df**	**p**	**AIC**
CHOLESKY	ACE	32.95	30	0.33	-27.05
	**AE**	**33.35**	**40**	**0.76**	**-46.65**
	CE	65.82	40	0.01	-14.18
INDEPENDENT	ACE	38.98	36	0.34	-33.02
	**AE**	**40.82**	**44**	**0.61**	**-47.18**
	CE	72.03	44	0.01	-15.97
COMMON FACTOR	ACE	49.68	42	0.19	-34.32
	**AE**	**49.68**	**47**	**0.38**	**-44.32**
	CE	81.49	47	0.00	-12.51

For each submodel, the chi^2 ^statistic, the degrees of freedom (df) the probability (p) and the Akaike information criterion (AIC) are shown. AIC is used to evaluate the fit, with the best fitting submodel shown in bold in each case.

**Table 5:** Genetic correlations (bold type, below in table) and unique environmental correlations (above) of the traits

	**HPT**	**RP**	**MIG**	**CAD**
**HPT**		0.29	0.01	0.17
**RP**	**0.20**		0.00	0.05
**MIG**	**0.23**	**0.36**		0.00
**CAD**	**0.35**	**0.56**	**0.65**	

HPT represents hypertension, RP Raynaud's phenomenon, MIG migraine, CAD coronary artery disease

## Discussion

This is the first study to examine the role of genetic and environmental factors in explaining the association between HPT, RP, migraine and CAD. The results suggest that all four variables share a heritable basis. These conditions have been shown to be associated with one another and individually each is known to have a genetic basis. The data presented here confirm these previous findings and suggest that shared environmental factors such as diet and lifestyle do not contribute to their expression. In view of the nature of these phenotypes, we speculate that the shared genetic component leads to a predisposition to vasospasm. Indeed, a 'vasospastic phenotype' to account for their co-occurrence has been postulated by others [14]. The demonstration of a single genetic component lends weight to such a theory.

A number of considerations should to be taken into account when interpreting these results. Self administered questionnaires were used for trait ascertainment, introducing the possibility of recall bias. In the present study efforts were taken to minimise recall bias: when surveyed, the twins were unaware of the hypothesis being tested; the questions relating to vascular phenotypes were included non-sequentially and were amongst many other questions in two questionnaires mailed at different times; and the twins completed questionnaires separately with no knowledge of their co-twin's responses. Furthermore there is no reason to suspect differential rates of recall in MZ when compared to DZ twins, hence any effects of recall should not have biased our estimates of genetic influence. Some conditions, such as RP, are notoriously difficult to diagnose regardless of the method employed [15] and it is possible that some subjects have cardiac valve rather than coronary artery disease. No attempt was made to differentiate primary RP and essential HPT from their secondary forms. However, questionnaire diagnoses were based on standard methods [8-10]. Despite these limitations, the traits' prevalences are in keeping with the findings of others for RP (9.6%[3], 5% and 16.8% according to geographical area sampled [16]), migraine (20% [17]) and CAD (8% [18]). In addition, heritability estimates are consistent with published findings for MIG (49–58% [19]), blood pressure (40–70% [20], 57% [21]) and CAD (15–30% [18]). Taken together, these observations suggest that our twins are representative of the adult female population and do not simply reflect a 'healthy volunteer' sample.

The assumptions underpinning twin studies themselves may be questioned. Unequal sharing of the family environment by MZ and DZ twins has been raised as a concern, but this has been shown not to be the case [22]. In addition, twins from this cohort have been shown to be similar to singletons with respect to anti-hypertensive drug use, blood pressure and other phenotypes [23]. The proposed inverse relationship between birth weight and HPT [24] and CAD [25] could potentially bias a study of twins and cardiovascular disease. The explanation for this relationship is still debated, but maternal environment has been suggested to be the main influence on adult blood pressure [26]. As we have demonstrated that the shared environment makes no significant contribution to the vasospastic phenotype, this is not a likely source of error.

It is clear that genetic factors play an important role in all four conditions. The demonstration that they are heritable is consistent with numerous reports of clustering in twin and family studies conducted in a range of settings, including a Dutch kindred with an autosomal dominant condition characterised by vascular retinopathy, migraine and Raynaud's phenomenon [27]. The detection of a common, genetically determined mechanism that contributes to these conditions is important because it points to an intermediate phenotype, vasospasm, and provides a possible focus for future studies. As with all chronic diseases and traits, the difficulty is in establishing which genes are responsible. Vascular tone is controlled at many levels, including local soluble mediators and neurotransmitters. Genes proposed through the study of the individual phenotypes include the beta2-adrenergic receptor gene in hypertension [28]; the muscle acetylcholine- and serotonin receptor genes in RP [29]; and a G protein subunit polymorphism and endothelial nitric oxide gene polymorphism in CAD [30]. A gain of function mutation in a vasoconstrictor or a loss of function mutation in a vasodilator may predispose to vasospasm: many of these genes deserve further consideration with the identification of a common genetic factor underlying HPT, migraine, RP and CAD.

In summary, this twin study has identified phenotypic associations between four vascular conditions and shown that the association is explained by a single common genetic factor. These findings are consistent with the proposed 'vasospastic phenotype' and suggest that studies of genes controlling vascular tone will help to define the genetic basis of these conditions.

## Competing interests

The author(s) declare that they have no competing interests.

## Authors' contributions

FMKW analysed the data and drafted the manuscript. LFC designed the questionnaires, analysed the data and assisted with the manuscript. TDS collected the twins on the Twin Register and assisted with the manuscript. AJM conceived of the study, collected the twins on the Twin Register, analysed the data and co-drafted the manuscript.

## Pre-publication history

The pre-publication history for this paper can be accessed here:


